# Collective Behaviors of Isotropic Micromotors: From Assembly to Reconstruction and Motion Control under External Fields

**DOI:** 10.3390/nano13212900

**Published:** 2023-11-03

**Authors:** Kai Feng, Ling Chen, Xinle Zhang, Jiang Gong, Jinping Qu, Ran Niu

**Affiliations:** 1 Key Laboratory of Material Chemistry for Energy Conversion and Storage, Ministry of Education, Hubei Key Laboratory of Material Chemistry and Service Failure, Hubei Engineering Research Center for Biomaterials and Medical Protective Materials, Semiconductor Chemistry Center, School of Chemistry and Chemical Engineering, Huazhong University of Science and Technology, Ministry of Education, Wuhan 430074, China; kfeng086@hust.edu.cn (K.F.); d202280208@hust.edu.cn (L.C.); m202270354@hust.edu.cn (X.Z.); jpqu@hust.edu.cn (J.Q.); 2 Key Laboratory of Polymer Processing Engineering, Ministry of Education, Guangdong Provincial Key Laboratory of Technique and Equipment for Macromolecular Advanced Manufacturing, National Engineering Research Center of Novel Equipment for Polymer Processing, School of Mechanical and Automotive Engineering, South China University of Technology, Ministry of Education, Guangzhou 510641, China

**Keywords:** isotropic micromotors, microswarms, collective behavior, motion control, complex environment

## Abstract

Swarms of self-propelled micromotors can mimic the processes of natural systems and construct artificial intelligent materials to perform complex collective behaviors. Compared to self-propelled Janus micromotors, the isotropic colloid motors, also called micromotors or microswimmers, have advantages in self-assembly to form micromotor swarms, which are efficient in resistance to external disturbance and the delivery of large quantity of cargos. In this minireview, we summarize the fundamental principles and interactions for the assembly of isotropic active particles to generate micromotor swarms. Recent discoveries based on either catalytic or external physical field-stimulated micromotor swarms are also presented. Then, the strategy for the reconstruction and motion control of micromotor swarms in complex environments, including narrow channels, maze, raised obstacles, and high steps/low gaps, is summarized. Finally, we outline the future directions of micromotor swarms and the remaining challenges and opportunities.

## 1. Introduction

Collective behavior is the autonomous organization of a variety of individuals to form a functional group, which widely exists in nature and ranges in all scales, including crystal molecules, bacteria colonies, macroscopic flocks of animals (fishes, ants, birds, etc.), and galaxies [1,2,3,4]. Compared to the individuals, the collective assembly of natural individuals could be more efficient in working together to accomplish complex tasks [5]. For example, schools of fish can move in an orderly manner or change shape and internal structure suddenly to adapt to their surroundings [6], collectives of ants can stack themselves to build bridges and transport heavy foods together [7], and bacterial colonies are efficient in accessing new sources of nutrients for increasing community size [8]. Similar to natural collective assemblies, artificial systems consisting of simple isotropic units assemble through physical/chemical field-induced interactions, and provide model systems to understand complex collective systems [9,10,11,12,13,14].

**Scheme 1:**
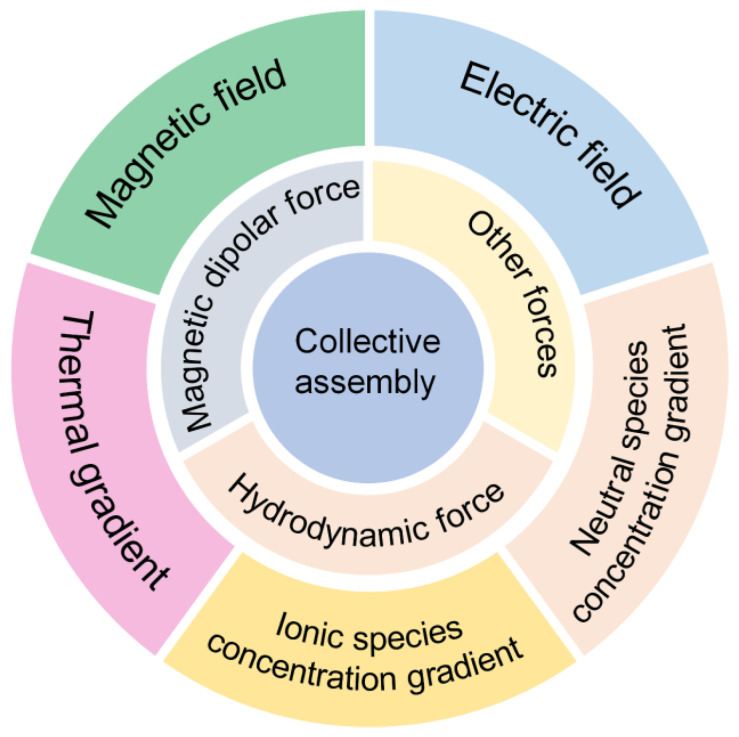
Collective assembly of isotropic micromotors via magnetic/hydrodynamic forces under different physical/chemical stimuli.

Self-propelled synthetic micromotors are capable of converting the surrounding energy into mechanical motion in fluidic environments, and give a new insight into the understanding of living matter and out-of-equilibrium systems [15,16,17,18,19,20,21]. To break the Scallop rule in a low Reynold regime, the symmetry of micromotors needs to be broken. Typically, Janus micromotors or micromotors of various shapes such as nanorod, nanosphere, core–shell nanowire, and nanotube are designed [18,22,23,24,25,26,27,28]. Among them, isotropic colloidal motors are easy to prepare and can self-assemble into large swarms for the transport of loads, which demonstrates the advantages of collective control [29,30,31]. To date, different reactions and external fields have been applied to generate colloid swarms (Scheme 1), including ionic/neutral species concentration gradients produced by a catalytic or noncatalytic reaction [32], thermal gradients generated by a photothermal conversion [33], and magnetic and electric fields [34,35]. These chemical reactions or external fields can affect microscopic forces among colloidal particles, such as the hydrodynamic force [36], magnetic dipolar force [37], π-π stack force [38], hydrophobic force [39], interfacial tension [40,41,42,43], etc., ultimately leading to the formation of microswarms. Unlike other micromotor swarms based on Janus particles and microtubes, the isotropic character of colloidal particles provides a model system to study the interparticle interaction and clustering mechanism in out-of-equilibrium systems, as well as contributing to larger colloidal clusters for drug delivery, resistance to external disturbance, and high efficiency imaging [44,45,46,47,48,49,50,51,52].

In this minireview, we summarize the new phenomena and processes of collective assembly of isotropic active colloids, and the reconstruction and motion of the micromotor swarms under external stimuli, and discuss the related fundamental principles and interactions. Then, we discuss the currently developed strategies for the motion control of micromotor swarms in complex environments to meet requirements in real applications. Finally, we outline the challenges and opportunities for the future development of microswarms.

## 2. Collective Assembly of Isotropic Micromotors

Constructing local fields plays an important role in the design of isotropic micromotor swarms. Because of the isotropy of individual micromotors, the field generated by single colloids cannot be converted to self-propulsion. However, the interactions between single colloids break the symmetry of fields and provide the driving force, such as magnetic forces, hydrodynamic forces, and other forces, for the formation of microswarms [53,54]. Thus, the collective assembly of micromotor swarms can be categorized according to the triggering field. Additionally, the interaction of these local fields has a key influence on the response of the microswarms to the environment or external stimuli. Understanding the coupling of local fields is necessary for realizing a reconfigurable assembly of isotropic micromotors. These local fields can be nonelectrolyte/electrolyte gradients induced by photo- or non-photo-triggered chemical reactions or externally applied stimuli. In this section, the design and assembly mechanisms of isotropic micromotor swarms are briefly described.

### 2.1. Chemical Gradient-Induced Micromotor Swarm

The chemical gradient field-induced collective assembly of micromotor swarms are usually triggered by a chemical reaction, including a catalytic reaction, ion exchange reaction, and self-dissociation reaction. The generated gradients of ionic/neutral species among individual colloids induce the diffusiophoresis, electrophoresis, and electroosmosis on a charged surface for the assembly of the colloids into a swarm.

#### 2.1.1. Photocatalytic Micromotor Swarms

Isotropic catalytic micromotors made of semiconductors tend to catalyze or react with surrounding molecules symmetrically, resulting in a uniform chemical gradient field around themselves. However, when multiple micromotors are in the proximity of each other, the generation of chemical gradients will be affected by each other on the basis of diffusiophoresis, electrophoresis, hydrodynamic resistance, etc., which can eventually be converted into a non-equilibrium chemical field inside and outside each cluster or swarm [29,55]. As a result, the swarm of isotropic micromotors exhibits multiple collective behaviors including self-assembly, reconstruction, and dispersion [56]. Among them, the photocatalysis of surrounding chemicals (fuels) becomes the main driving force to actuate the collective behavior of isotropic micromotors. In order to obtain light-controlled active micromotor swarms, the reactivity of micromotors, the physicochemical properties of catalyzed species (amount, valence state, diffusion coefficient, etc.), and the surface electrical properties of micromotors and substrates should be mainly evaluated.

**Figure 1:**
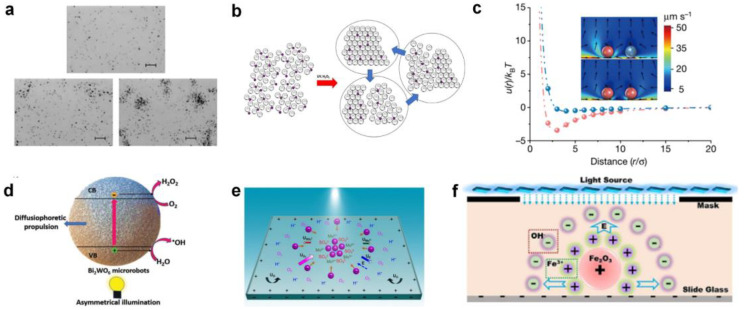
Photocatalytic micromotor swarms. (**a**) Collective behaviors of AgCl particles under UV exposure in deionized water. Reproduced from [56]. Copyright 2009 Wiley-VCH GmbH. (**b**) Self-assembly of silica microspheres in the presence of silver phosphate micromotors under UV light in hydrogen peroxide solution. Reproduced from [57]. Copyright 2020 Wiley-VCH GmbH. (**c**) Phoretic flow field and effective potential of phase-segregated photosensitive TiO_2_ colloidal particles. Reproduced form [58]. Copyright 2023 Springer Nature. (**d**) Motion and swarming mechanism of Bi_2_WO_6_ microrobots. Copyright 2020 Wiley-VCH GmbH. Reproduced from [30]. (**e**) MoS_2_ colloidal motors (pink particles) undergoing self-diffusiophoresis on a negatively charged substrate to form a swarm. Under illumination of UV light, MoS_2_ colloid motors at the center of the light consume O_2_ and generate cations and anions to induce an electric field (*E*) pointed inwards (blue arrow). Reproduced from [59]. Copyright 2021 Wiley-VCH GmbH. (**f**) Mechanism of light-induced electrolyte diffusiophoresis caused by Fe_2_O_3_ nanomotors with light-irradiation-consuming H_2_O_2_ molecules. Reproduced from [60]. Copyright 2019 Wiley-VCH GmbH.

Up to now, many semiconductor-based micromotor swarms have been designed. For example, Ibele et al. [56] reported micrometer-sized silver chloride (AgCl) particles that can secrete H^+^ and Cl^−^ ions under UV illumination in deionized water (at pH 5). Moving by self-diffusiophoresis, each AgCl particle responds by “schooling” into regions with higher particle concentrations (Figure 1a). Moreover, the photo-inactive silica particles can respond to the ion secretion of AgCl by swimming towards and surrounding individual AgCl particles. Altemose et al. [57] reported the oscillatory waves generated by silver phosphate micromotors which lead to the hexagonal packing of inert silica under UV light and H_2_O_2_ (Figure 1b). The colloidal crystals can form/relax under/without UV illumination. The electroosmotic flow generated on the charged substrate dominates the motion and assembly/dispersion of all particles in the system. Zheng et al. [58] recently developed commercial dye-stained TiO_2_ microspheres to realize phase segregation and interaction tuneability in response to the light of different wavelengths (Figure 1c). At the basis of the photoexcitation of dyes, the redox reaction on TiO_2_ particles generates chemical gradients, which results in diffusiophoretic flow and effective attractive potential between each other. The particle–particle interaction results in selectively activated colloidal swarms. Villa et al. [30] exhibited self-propelled perovskite-like bismuth tungstate (Bi_2_WO_6_) micromotors that can be activated in water under visible-light irradiation (Figure 1d). The activation mechanism involves the generation of electron-hole pairs that react with water and O_2_ to produce hydroxyl radicals (•OH), protons (H^+^), hydroxyl groups (OH^−^), and H_2_O_2_ species. The chemical concentration gradient around the spherical microparticles contributes to the swarming behavior of Bi_2_WO_6_ micromotors. Chen et al. [59] reported UV light-activated MoS_2_ colloidal motors which can generate H^+^, SO_4_^2+^, and Mo^6+^ via a photo-corrosion reaction as the surface of MoS_2_ is oxidized into MoO_3_ under exposure to UV light (Figure 1e). They found that the collective motion of MoS_2_ could occur on both negatively charged and positively charged surfaces, which verifies that the diffusiophoresis induced by the locally consumed oxygen gradient dominates the phototaxis of colloidal motors.

In addition to photoexcited semiconductor materials, micromotors based on Fenton-like reactions are also capable of generating ionic concentration gradients that promote spontaneous self-assembly when stimulated by external light. For example, Zhou et al. [60] employed Fe_2_O_3_ nanoparticles as self-assembly units which can react with H_2_O_2_ under visible light via a photo-Fenton reaction (Figure 1f). In this catalysis process, ferric ions and hydroxide ions, being different in diffusion coefficients, are released from the surface of the nanomotors. Then, a diffusion-induced electric field is established, which acts not only on the nanomotors, but also on any charge surfaces. As a result, electroosmotic flow is generated to drive the nanomotors towards each other to form the desired shape based on the illumination.

**Figure 2:**
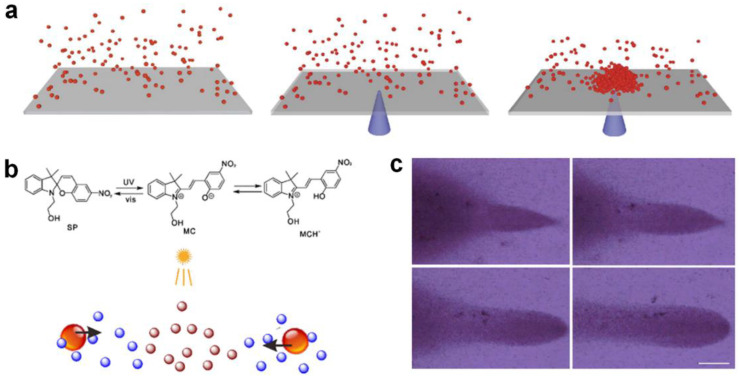
Assembly by environmental matter catalysis. (**a**) Assembly of poly(12-hydroxy-stearic acid) (PHSA)-stabilized poly(methyl methacrylate) (PMMA) colloids on the coupling of electromagnetic illumination to a ITO-coated glass substrate. Reproduced from [61]. Copyright 2014 Springer Nature. (**b**) Photoisomerization causing chemical gradients under UV spot irradiation. (**c**) Aggregated silica particles following a moving UV spot. Reproduced from [36]. Copyright 2020 ACS publication.

Catalytically or photo-chemically consuming environmental chemical compounds can also lead to microparticle agglomeration. This micro-nano cluster assembly method does not depend on certain specific active micro-nanoparticles; it therefore greatly expands the range of candidates for the formation of micromotor swarms, and can be used to assemble chemically inert micro-nano particles. Kim et al. [61] reported the photo-induced assembly of PMMA particles on conductive or semi-conductive substrates (for example, ITO- or gold-coated substrates) under the illumination of laser of varying wavelengths (Figure 2a). They found that at the basis of illumination, a local electric field could be established on an absorptive, conductive substrate such as ITO to direct colloid motion and lead to colloid assembly. The photo-induced assembly is strongly affected by solvent conditions and the surface functionalization of PMMA particles, indicating the electrophoresis-dominated assembly process. Wu et al. [36] investigated the transport of aggregated silica microspheres via the photoisomerization of spiropyran in solution (Figure 2b). Under the illumination of UV spot sources, the silica microspheres moved and aggregated at the center of the light spot and finally formed three-dimensional aggregates (Figure 2c). The researchers found that the spiropyran concentration as well as the chemical gradient derived from UV irradiation play key roles in silica microsphere aggregation. Based on the strength of osmotic flow, other inert colloidal particles such as PS microspheres can also be transported to the light spot.

#### 2.1.2. Non-Catalytic Micromotor Swarms

**Figure 3:**
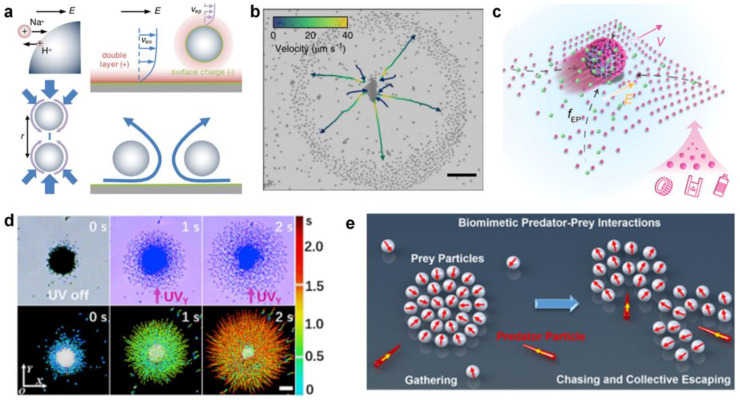
Non-catalytic reaction-triggered micromotor swarms. (**a**) Clustering and phototactic motion behaviors of TiO_2_ micromotors with high-surface acidic bridging hydroxyls. Reproduced from [31]. Copyright 2017 PRL. (**b**) Schematic demonstration of biomimetic predator-prey interactions based on the binary repulsive−attractive diffusiophoretic interactions. Reproduced from [63]. Copyright 2021 Springer Nature. (**c**) Long-range interaction between ion-exchange resin colloids through electroosmotic flow. Reproduced from [64]. Copyright 2022 AAAS. (**d**) The ion-exchange interaction of the ZnO-sulfonated PS complex. Reproduced from [32]. Copyright 2019 ScienceDirect. (**e**) Self-driven magnetorobot (SMR) consisting of ion-exchange resin microsphere-enabling adsorption of MNPs from the aqueous environment. Reproduced from [65]. Copyright 2020 ACS Publicaton.

The above research on light-triggered swarms greatly improves the understanding of the formation mechanism of micromotor clusters, and lays a foundation for constructing single-component isotropic micromotor swarms with an on-off control. While the non-catalytic reaction-triggered swarms often involve spontaneous interactions of multiple components in a system, inducing complex phase behaviors that exceed those of single-component clusters, such as predator–prey interactions. This multi-component phase separation often results from the exchange of ions between each other. Michelin et al. proved that asymmetries in geometry are sufficient to induce chemical gradients and swimming [62]. A typical example is ion-exchange resin particles, which can exchange cations for protons and generate pH gradient and long-rang electroosmotic flow. Niu et al. [31] first demonstrated the long-range flow and its role in acting as a long-range attractive interaction between two particles, which induces the self-assembly of colloidal molecules (Figure 3a). Later on, the Tang group [63] demonstrated the “waste” ion exchange-induced quorum sensing of the ZnO-sulfonated PS complex (Figure 3b). Briefly, the ZnO nanorod serves as a Zn^2+^ source for the ion exchange of sulfonated PS, and the H^+^ released from the sulfonated PS accelerates the dissolving of ZnO in turn. The migration speeds of the system are greatly enhanced where the two active particle species are regulated by the nearby counter particles. At an equilibrium position of the chemotaxis attraction and surface slipping flow, the ZnO-sulfonated PS complex is assembled. Then, the researchers explored the application of the long-range attractive flow in microplastic removal in nonmarine water (Figure 3c) [64].

Another example is anatase TiO_2_ micromotors with abundant hydroxyl groups. Mou et al. [32] demonstrated that the surface hydroxyl groups on TiO_2_ can help to secrete H^+^ ions from surface acidic bridging hydroxyls and OH^−^ ions from basic terminal hydroxyls by dissociating water (Figure 3d). Then, a converging electroosmotic flow was established to drive the aggregation of particles due to the migration of cations in the electrical double layer of micromotors and glass substrate. However, when UV light was applied, the TiO_2_ flocks catalyzed environmental H_2_O_2_ to produce a cloud of O_2_ molecules around the micromotors, which induced the dilatational phototaxis. Recently, Mou et al. [29] demonstrated that the micromotor predator–prey system, containing diffusiophoretic repulsive and attractive micromotors, could be established without additional chemical fuels (Figure 3e). The two species of microparticles, including active Ag_3_PO_4_-TiO_2_, ZnO-AgBr, and ZnO-TiO_2_ micromotor systems, show dynamic group reconfigurations based on local repulsion.

### 2.2. Photo-Thermal Effects-Caused Self-Assembly

By utilizing various thermal forces such as thermophoresis, photophoresis, and convective flows in a light-controlled temperature field, researchers have endowed micromotors with versatile manipulation methods. The thermophoresis-actuated micro/nano motors and swarms have been used in the fields of nanomedicine, chemical and biological sensing, and micro patterns [65,66]. Here, the heat-mediated assembly of micromotors and its formation mechanism are briefly introduced.

**Figure 4:**
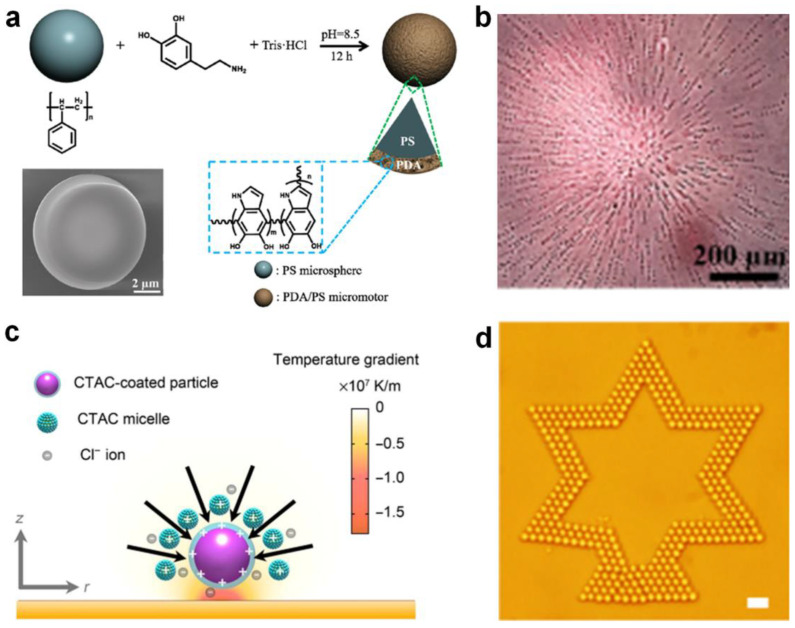
Self-assembly by photothermal conversion-induced thermal gradient. (**a**) Fabrication and (**b**) optical microscopic image of NIR light-driven positive phototaxis of PS@PDA micromotors. Reproduced from [33]. Copyright 2019 ACS Publication. (**c**) Light-directed thermoelectric field around colloid particle and (**d**) 2D assembly of a star pattern of 2-μm silica beads. Reproduced from [67]. Copyright 2017 AAAS.

Sun et al. [33] demonstrated a novel micromotor consisting of a polystyrene microsphere core and a polydopamine shell (PS@PDA) which can be activated by NIR light (Figure 4a). Interestingly, the micromotor demonstrates a concentration-dependent motion direction reversal, i.e., a single micromotor exhibits negative phototaxis, whereas a group of micromotors shows positive phototaxis-induced aggregation near the NIR light spot, which is tunable in shape via the light irradiation position (Figure 4b). The differences can be attributed to the competition between the thermophoretic force and hydrodynamic drag caused by the thermal buoyancy. Lin et at. [67] demonstrated that ionic surfactant cetyltrimethylammonium chloride (CTAC) micelles can be used as positive macro ions to create a thermoelectric field for the optical trapping of colloidal particles (Figure 4c). They found that the added-CTAC molecules and micelles would adsorb onto the colloidal particles through electrostatic interaction and/or hydrophobic interaction. Then, a temperature gradient along the z axis was established through the irradiation of a laser beam, which could drive both the CTAC micelles and Cl^−^ ions to the cold side. As a result, a thermoelectric field was established which can manipulate the assembly of colloidal particles into two-dimensional (2D) colloidal matter (Figure 4d). In this way, colloids of various sizes and materials can be efficiently assembled, which is applicable to a range of particles.

### 2.3. Magnetic Field-Induced Self-Assembly

Magnetism, as an external stimulus, is commonly employed to provide additional magnetic dipole–dipole force beyond the traditional electrostatic, gravity, and hydrodynamics forces for the control of micromotors. The advantages of applying a magnetic field force include the capacity for high temporal and spatial control, and superior non-invasive and harmless characteristics. Moreover, the magnetic field can promote the formation of micromotor swarms that is easy to control. Up to now, a large number of studies on the swarm behaviors of magnetic micromotors have been reported.

**Figure 5:**
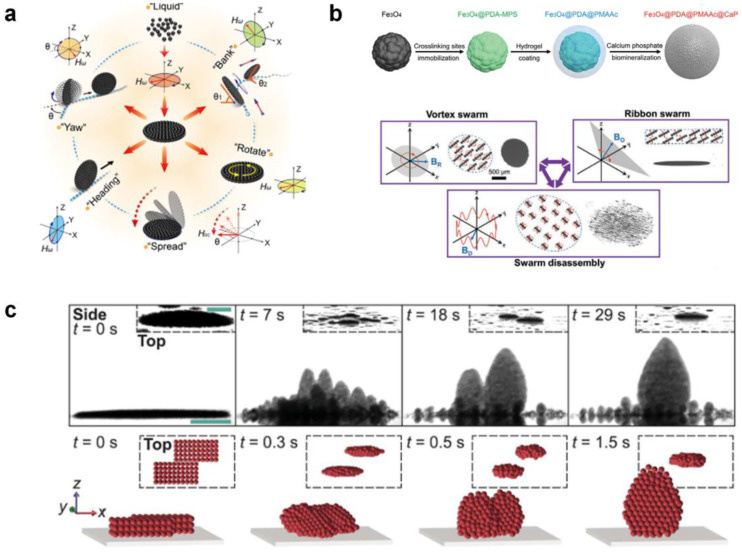
Self-assembly by external magnetic fields. (**a**) Magnetic-driven wheel-like microswarm (WLM) of multiple switchable motion modes. Reproduced from [68]. Copyright 2022 ACS publication. (**b**) Fabrication and manipulation of magnetic colloidal microswarms with three kinds of collective behaviors. Reproduced from [34]. Copyright 2021 Wiley-VCH GmbH. (**c**) Magnetic generation of vertical collectives from dispersed colloidal particles. Reproduced from [69]. Copyright 2022 AAAS.

Li et al. [68] reported a ferromagnetic nanoparticle-assembled wheel-like micromotor swarm which maintains dense and stable morphology (Figure 5a). They employed a “packing under rolling” method to assemble the micromotor swarms with variable aspect ratios via integrating AC and DC magnetic field control. The frequency and strength of the external magnetic field were tuned to fabricate swarms and to achieve five switchable motion modes (Figure 5b). Moreover, the micromotor swarms can navigate along the intestinal inner wall with controllable direction, intending to repair microscale intestinal perforation in the future. Jin et al. [34] fabricated core–shell-structured paramagnetic nanoparticle@ calcium phosphate (Fe_3_O_4_@PDA@PMAAc@CaP) to achieve magnetic swarm control and targeted drug delivery (Figure 5c). The swarm patterns of Fe_3_O_4_@PDA@PMAAc@CaP could be reconfigured by changing the types of superposed magnetic fields in a reversibly way. Moreover, by programming the external magnetic fields, the swarms can serve as the drug delivery vehicle with on-demand cargo loading and release. Law et al. [69] conducted pioneering work in presenting a strategy to overcome the self-assembly of dynamic colloidal structures along the vertical direction via a combination of dipole–dipole interactions, gravity, and hydrodynamic drag forces. The 3 μm diameter paramagnetic particles with hydrophilic surface were suspended in 0.1% Tween 20 solution to prevent nonspecific aggregations. The dual-axis-oscillating magnetic field was applied to assemble magnetic particles against gravity into vertical collectives. Moreover, the researchers found that the self-assembled swarms can perform controllable changing in shape, orientation, inclination, and locomotion, which can be used to mimic ant colonies in multi-dimensional scales.

### 2.4. Electric Field-Caused Self-Assembly

**Figure 6:**
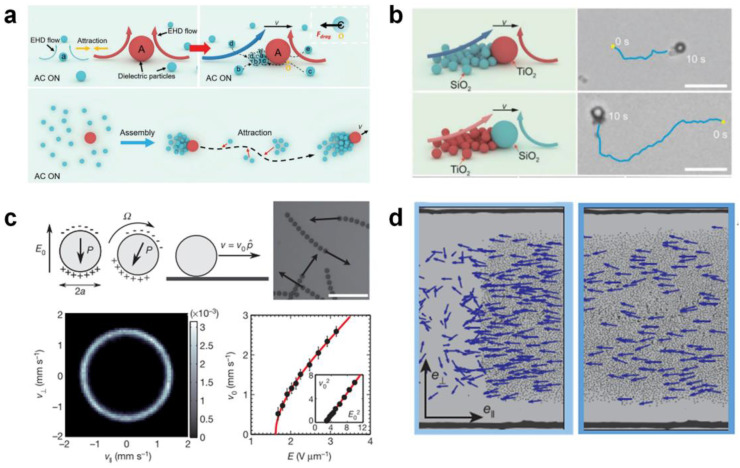
Self-assembly in external electric fields. (**a**) Schematic illustration of the assembly and (**b**) actuation of hierarchical micromotor swarms by applying AC electric field. Reproduced from [54]. Copyright Wiley-VCH GmbH. (**c**) Population mechanism of self-propelled colloidal rollers and (**d**) the formed macroscopic band propagating along the racetrack via electric polarization. Reproduced from [35]. Copyright 2013 Spring Nature.

Electric fields can trigger swarm behaviors based on dipole–dipole interactions, dielectrophoresis forces, and hydrodynamic interactions. Liang et al. [54] reported a self-organized swarm of dissimilar microparticles by applying AC electric field (Figure 6a). The dissimilar dielectric microparticles can self-assemble into leader–follower-like microswarms hierarchically in an AC electric field, due to the unbalanced surrounding local electrohydrodynamic (EHD) flows generated around different microparticles and diffusiophoretic interactions. Moreover, the microswarms can exhibit reversible states between stop and go in response to vertical UV irradiation, which may open remarkable opportunities in biomedical intelligent microswarms (Figure 6b). Bricard et al. [35] reported self-organized colloidal roller populations which show coherent motion in a unique direction (Figure 6c). The unstable charge distribution at the sphere’s surface above a critical electric field induces a spontaneous symmetry breaking and a net electrostatic torque, leading to the well-known Quincke rotation. The researchers suggested that the hydrodynamic interactions between Quincke rollers lead to the emergence of collective motion (Figure 6d).

## 3. Navigation in Complex Environments

Traditional investigation into microrobotic swarms has mainly focused on flat surfaces. However, in real-application environments, such as the human body, micromotor swarms need to face a variety of complex environments such as curved and narrow channels, complex mazes, raised obstacles, and high steps and low gaps. The reconfiguration and task sharing of the micromotor swarms on the demands of the environment is highly required for a variety of applications. In this section, the adaptive navigation of micromotor swarms in different scenarios is briefly described.

### 3.1. Curved and Narrow Channels

**Figure 7:**
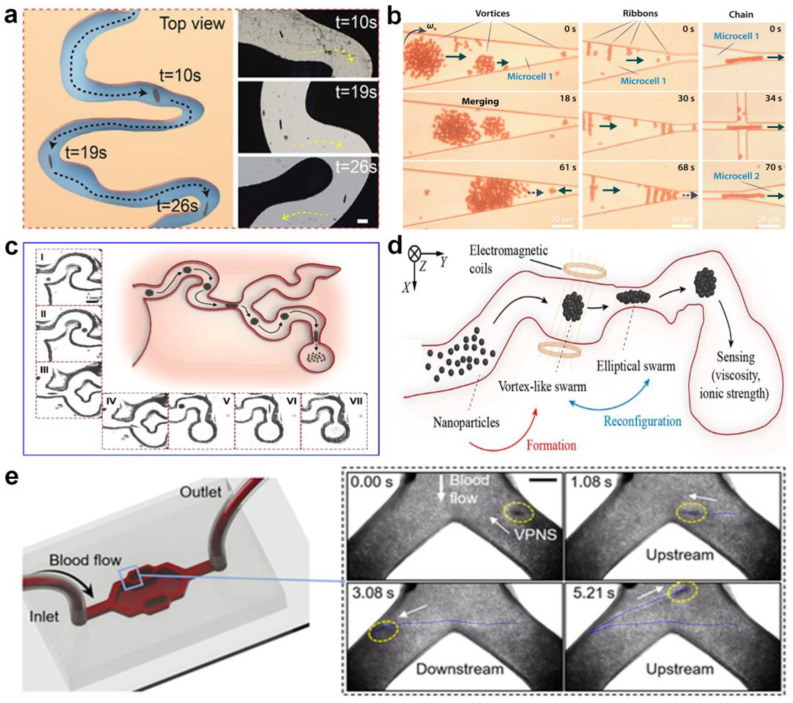
Locomotion in curved and narrow channels in plane. (**a**) The WLM passes through curved channels. Reproduced from [68], copyright 2022 ACS Publication. (**b**) Comparison of the different organizations of microrobotic swarms in passing through narrow channels. Reproduced from [45], copyright 2019 AAAS. (**c**) Microswarms traverse a variety of circuitous, branching, and winding channels. Reproduced from [34], copyright 2021 Wiley-VCH GmbH. (**d**) Colloidal microswarms navigate through curved and narrow channels for the sensing of viscosity and ionic strength in the targeted region. Reproduced from [70], copyright 2022 ACS Publication. (**e**) VPNSs travel freely in complex simulated 3D vessels. Reproduced from [71], copyright 2023 AAAS.

Adapting microrobotic swarms to closed optimized environments without compromising their integrity and functionality is a critical step towards practical applications. To test the dynamic adaptability of microrobotic swarms to environmental constraints, microrobotic swarms have been designed and guided through a series of curved and narrow channels. For example, Li’s group [68] proposed a wheel-like magnetic-driven microswarm (WLM) that imitates a band-aid. By integrating AC and DC magnetic field control, multiple switchable motion modes “yaw”, “bank”, “rotate”, “spread”, and “heading” have been realized. Interestingly, in “bank” mode, the WLM swings around the direction of advance and maintains rolling at an oblique angle, thus enabling the WLM to pass through the curved channel easily and rapidly (Figure 7a). As for narrow channels, He’s group [45] used an alternating magnetic field to program hematite colloidal particles into liquid, chain, vortex, and ribbon-like microrobotic swarms, and enable fast and reversible transformations between them. Interestingly, only the chain could efficiently pass the narrow channel (Figure 7b). Although the speed of movement was slightly reduced because of the drag torque from the microfluidic walls, the chain’s collective formation could be well maintained because of its shape conforming to the narrow channel.

However, when curved channels with narrow passes are present, both structural reconfiguration and locomotion direction control are needed to avoid the restricting channel walls, which are highly demanding and need the fast response of the micromotor swarms. Normally, the multiple collective states could be reproduced with ease in the same active matter system by a facile programming of the magnetic fields. In a group of paramagnetic nanoparticles, the transition between rotating and oscillating magnetic fields could trigger the reversible transformation between vortex- and ribbon-like microswarms. Because the former type possesses rapid and dexterous locomotion capabilities while the latter could pass through narrow channels with its slender body, such magnetic active matter has high environmental adaptability through actively switching between different collective states (Figure 7c) [34]. Similarly, Yu’s group showed that the suspended paramagnetic nanoparticles are gathered into a vortex-like swarm first, and then actuated along the channel in a controlled manner (Figure 7d). When the swarm encounters a narrow channel, an elliptical pattern is transformed to pass through the environment. The pattern reconfiguration is then performed again to convert the swarm back [70].

Reversible transformation between vortex-like swarms (VPNSs) and elliptical swarms (EPNSs) can also be triggered by applying programmed elliptical magnetic fields. As reported by Zhang’ group, both VPNSs and EPNSs can effectively perform reversible contraction and elongation to adaptively navigate curved and narrow channels [72]. Notably, in a sharp channel, the swarm can be transformed into a VPNS to cross the channel. And when the VPNS approaches the right half of the channel with the narrowed space, it performs the pattern reconfiguration into an EPNS and passes through in order to avoid hitting the wall. After the swarm exits the channel, it can revert to a VPNS again.

Unlike previous reports, Feng’s group [71] transformed MNPs from a loose suspension state into a swarm that combines liquid deformability and solid robustness by applying an innovative three-dimensional (3D) tilted rotating magnetic field. In the complex simulated 3D vessels, the VPNS can advance and dock toward the lateral sides of the channel under the control of the magnetic field, and travel freely between branches according to a preset trajectory (Figure 7e).

### 3.2. Complex Mazes

In the process of executing tasks, microrobotic swarms not only need to cross the narrow/curved channels in the road, but also to choose the correct route among numerous intersections to effectively reach the destination. Therefore, the locomotion of microrobotic swarms in maze environments has been explored. For example, He’s group. [73] designed a snakelike magnetic microrobot swarm (SMS) assembled from peanut-shaped hematite colloidal particles driven by a rotating magnetic field, which exhibits a dynamic-equilibrium chain that can capture additional microrobots to complete its evolution. By regulating the rotational frequency, intensity, and steering angle of the input magnetic field, the SMS can achieve high-precision trajectory tracking and serpentine locomotion in an unknown micromaze without collision with the wall. Differently, Sun et al. [74] designed a magnetic tweezer system that assembles low-density magnetic microparticles to form a stable and compact microswarm at a predetermined position. The actuation of the magnetic tweezers enables the precise navigation of the microswarm in an open micromaze without relying on real-time image feedback. More interestingly, the swarm could successfully navigate to the targeted position even if the micromaze is covered.

**Figure 8:**
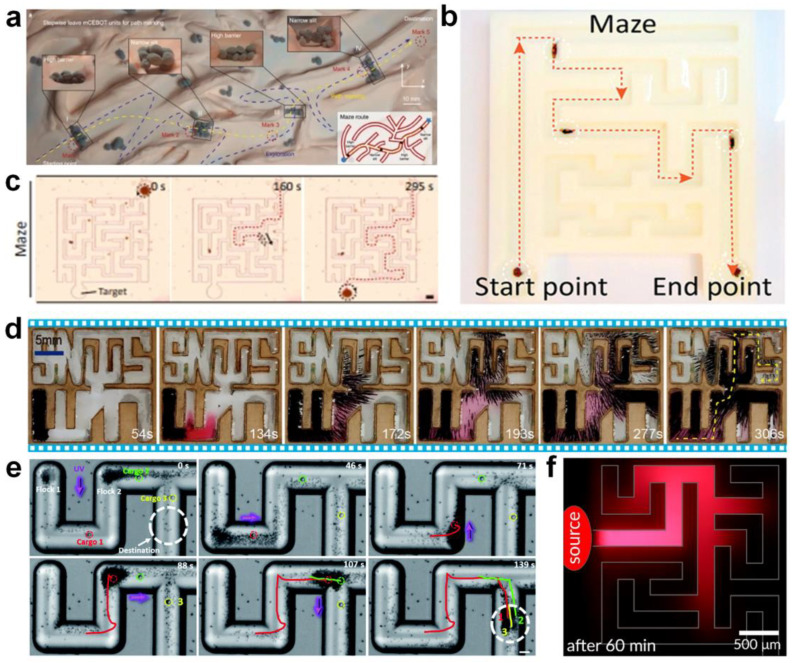
(**a**) An mCEBOT traverses a complex maze. Reproduced from [75], copyright 2022 Nature Publishing Group. (**b**) MUCR@MLMD navigation in a complex maze. Reproduced from [76], copyright 2012 Wiley-VCH GmbH. (**c**) A standing RSM passes through a complex maze. Reproduced from [77], copyright 2021 Wiley. (**d**) Tentacle-like microswarms navigate a narrow and twisting micromaze. Reproduced from [78], copyright 2022 ACS Publication. (**e**) TiO_2_-MFs are guided by UV light to pass through the “*S*” maze. Reproduced from [79], copyright 2021 Royal Society of Chemistry. (**f**) Mazing solving of chemotaxis droplet swimmers. Reproduced from [80], copyright 2017 PNAS.

For navigation in maze environments, it is particularly important to shorten the time for the microrobotic swarm to pass through the maze. Yang et al. [75] reported a milli-scale cellular robot (mCEBOT), which can selectively assemble (e.g., end-by-end and side-by-side) into diverse morphologies under the magnetic field to adapt to the unstructured environments. Benefiting from the heterogeneous assembly-endowed selective attaching and detaching of units, mCEBOT can be used for unstructured environment exploration and path marking by selective and stepwise unloading units, omitting the attempt of invalid paths and reaching the destination faster and more efficiently (Figure 8a). On the one hand, mCEBOT can easily overcome topographically complex mazes through the simultaneous reconfiguration of morphologies and tuning of motion behaviors.

However, for more complex maze environments, several collective state transitions are required for smooth passage. For example, in the case where the size of the swarm is larger than the width of the maze passage, the MUCR@MLMD swarm prepared by Sun et al. [76] automatically varies from a circular to a fusiform shape during movement to negotiate with the maze environment. Even affected by the inner wall of the passage, the swarm could consistently maintain a stable shuttle shape without dispersing, and finally reach the target area with almost no loss (Figure 8b). Similarly, Sun et al. [77] designed microswarms that exhibit both liquid-like and solid-like behaviors. By increasing the frequency of the rotating magnetic field and the magnetic field tilt relative to the x–y plane, the microswarms can transform from liquid-like to solid-like, while also changing from lying flat into a standing state. In this case, the standing swarm can easily pass through a maze much smaller than its own dimensions under the control of the magnetic field, and maintain stability during the process (Figure 8c). While in the narrow and twisted maze environment, Xu et al. [78] designed a tentacle-like reconfigurable microbot swarm by programming paramagnetic microparticles into reconfigurable carpets with numerous cilia. This wirelessly controlled microrobot swarm is constructed via a strong gradient magnetic field in combination with a programmable oscillating magnetic field. By applying the gradient magnetic field, the tentacle-like swarm can be reconfigured to deliver the drug to a hard-to-reach corner in a narrow and twisting micromaze with little effect on other locations (Figure 8d).

In addition to magnetic field control, light fields can also control microswarms to pass through complex mazes. For example, Zhang et al. [79] showed that the hydroxyl group-rich TiO_2_ micromotor swarms can be guided by UV-light to travel through tunnels via negative phototaxis. After turning the UV light to the +X direction, the merged TiO_2_-MF coercing the cargo moved toward the right corner. Using a similar method, the TiO_2_-MF was guided to pass through the “S” maze (Figure 8e). In addition, Maass’ group [80] presented a self-propelling droplet swimmer that exhibits negative autochemotaxis along micellar surfactant gradients, which is further used to guide the droplet travel through a microfluidic maze. Similarly, a depletion of empty micelles in the wake of a droplet swimmer causes negative autochemotaxis and thereby trail avoidance. The chemotaxis could potentially be used to guide the maze solving of micromotor swarms, which is, however, still unexplored (Figure 8f).

### 3.3. Raised Obstacles

**Figure 9:**
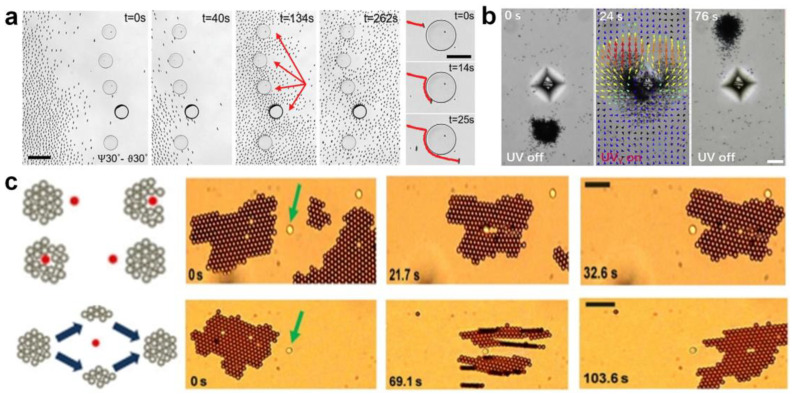
(**a**) A microswarm navigates through an array of obstacles by compression and expansion. Reproduced from [81], copyright 2019 Wiley−VCH GmbH. (**b**) A TiO_2_ micromotor swarm autonomously bypasses a prism obstacle under the guidance of UV light. Reproduced from [32], copyright 2019 ScienceDirect. (**c**) A colloidal carpet moves against an immobile colloid. Reproduced from [82], copyright 2015 American Physics Society.

Besides curves and narrow channels, micromotor swarms may also be confronted with obstacles on a flat surface, such as red blood cells in blood. To mimic the real-application environment, different obstacles were designed and used to explore the avoidance performance/strategy of micromotor swarms. For example, Sitti’s group [81] showed that a linear chain-like mobile microrobotic swarm can be guided by a processing magnetic field through an array of convex obstacles, with the swarm being compressed and expanded when contacting obstacles (Figure 9a). Notably, individual microrobots slid on the obstacle walls in a counterclockwise direction and, once the contact with the wall was lost, continued freely in the direction of magnetic propulsion.

However, in certain special circumstances, microswarms need to adapt the profile of the obstacle. For example, Mou et al. [32] reported microswarms formed by the spontaneous aggregation of TiO_2_ micromotors with rich hydroxyl groups. Under global UV navigation, the microswarms can migrate along pre-designed paths. When encountering a prism obstacle, a traveling swarm deforms into a V-shaped swarm and embraces the obstacle. Then, it splits into two subflocks to adapt the profile of the obstacle when it continuously moves forward. Immediately after passing the obstacle, the two subswarms re-join at the far side of the obstacle and continue to move phototactically as a whole (Figure 9b). Similarly, under a rotating field polarized in the x–z plane, a magnetic carpet assembled from monodisperse paramagnetic particles was designed by Tierno and co-workers. [82]. Interestingly, the carpet shows a similar behavior when navigating against an obstacle, i.e., an immobile microparticle (Figure 9c).

In addition to designing different microswarms, different algorithms have been proposed to plan paths and thus avoid obstacles. For example, Wang et al. [83] optimized in real time the pattern formed by magnetic droplets based on the genetic algorithm. Following the position of the magnetized needle, the navigation of dynamic patterns was demonstrated along preplanned paths under processing magnetic fields. At the same time, a particle swarm optimization (PSO)-based optimal path planner with an obstacle-avoidance capability was designed, which avoided obstacles by generating a path between the field boundary and obstacles. Inspired by the technique of radar detection, Liu et al. [84] proposed a radar-based control algorithm for dynamic obstacle avoidance using a microrobotic swarm. Based on the position and size of the swarm captured through imaging feedback, the hierarchical radar is generated. The detection circle, the safety circle, and the prediction circle of the radar cooperate to select the optimal moving direction of the swarm. In addition, combined with visual feedback, the swarm can be controlled to move to predefined targets with the interference of multiple dynamic obstacles.

### 3.4. High Steps and Low Gaps

**Figure 10:**
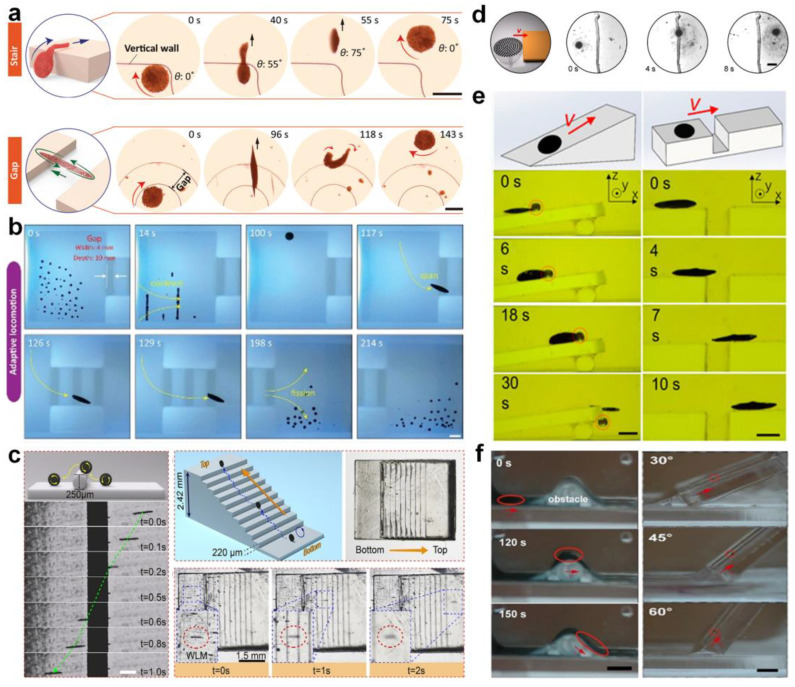
(**a**) An RSM climbs across a stair with a height of 40 μm and an FSM spans a gap with a depth of 40 μm. Reproduced from [77], copyright 2021 Wiley. (**b**) Dispersed ferrofluid droplets merging to form an entity and then crossing a continuous deep gully and step. Reproduced from [85], copyright 2023 Wiley-VCH GmbH. (**c**) In “heading” mode, the WLM climbs over cylindrical obstacles taller than itself and climbs a 10-step stair. Reproduced from [68], copyright 2022 ACS Publication. (**d**) A ROMS climbs across a stair with a height of 38 μm. Reproduced from [86], copyright 2022 Wiley-VCH GmbH. (**e**) A wheel-like swarm climbs slopes and strides across crevices in paired-fin motion mode. Reproduced from [87], copyright 2022 Frontiers Media S.A. (**f**) ROSrobot swarms climbing slopes of different inclines in a vertical magnetic field. Reproduced from [88], copyright 2022 Wiley-VCH GmbH.

To address multitasking requirements, microswarms not only need to adapt to the different environments mentioned above, but also to be able to overcome the obstacles of high steps and low gaps encountered during the movement process. In high-step and low-gap environments, more efforts must be made to break the limitations of two-dimensional motion and improve the maneuverability of microswarms. Here, inspired by viscoelastic fire ant aggregations, Sun et al. [77] proposed microswarms that exhibit both liquid-like and solid-like behaviors. By spatiotemporally programming an applied magnetic field, the microswarms can be reconfigured into four collective patterns in situ, i.e., a round liquid-like microswarm (RLM), a round solid-like microswarm (RSM), a fusiform liquid-like microswarm (FLM), and a fusiform solid-like microswarm (FSM), with reversible transformation between these patterns. Interestingly, an RSM can effortlessly cross a stair with a height of 40 μm, and an FSM can span a gap with a depth of 40 μm (Figure 10a). Similarly, Sun et al. [85] programed the magnetic torque to reconfigure active ferrofluid droplets into horizontal and vertical layer-upon-layer collectives. Via the spatiotemporal programming of the magnetic field, the horizontal self-assembled swarms exhibit vortex-like, chain-like, and crystal-like behaviors, while the vertical collectives exhibit a cascading morphology. It is worth noting that the chain-like collectives or crystal-like collectives cannot easily pass through wide gutters (gap: 4 mm) and steps (height: 4 mm), whereas the aggregation of dispersed droplets into an entity with a length of 8 mm can smoothly pass through the continuous gap and cross the stair (Figure 10b).

In addition to the combination of multiple collective modes, a single collective mode can also climb stairs. For example, Yue et al. [68] reported a wheel-like magnetically driven microswarm (WLM). When the magnetic field is controlled to move to “heading” mode, an enhanced rotating magnetic field is generated in the vertical plane, driving the WLM to roll towards the obstacle. Once in contact with the boundary of the obstacle, the WLM slightly deforms. Subsequently, a frictional force is generated on the contact surface opposite to the instantaneous velocity at the edge of the WLM. Under the action of frictional force, the WLM could successfully climb over the cylindrical obstacle. In addition, in “heading” mode, the WLM is also controlled to climb over a 10-step staircase with a height of 2.44 mm. The morphology of the WLM always remains a stabilized entity, despite suffering from a significant impact force when climbing over each step of the staircase (Figure 10c). Interestingly, the round-like magnetic microswarm reported by Zhang et al. can also effortlessly climb a stair with a height of 38 μm with the same force manner, with the difference that the round-like magnetic microswarm was horizontal (Figure 10d) [86]. Moreover, by setting the pitch angle and frequency of the magnetic field, the vortex swarm reported by Wang et al. was also able to easily climb over “cliffs” and go across gullies that are much taller than its diameter [89].

When encountering a slope, it is similar to climbing a stair. For example, a vector-controlled microswarm reported by Li et al. can be manipulated to stand vertically and swim like a wheel by adjusting the direction of the magnetic field plane [87]. Using a biomimetic design from the fin motion of fish, the plane of actuating magnetic field swings alternately in the positive y-axis direction, and the negative y-axis direction causes the wheel-like swarm to climb up the slope and cross the gully (Figure 10e). To demonstrate that a slope has little effect on motion, Zhao et al. [88] designed magnetically actuated ROS-scavenging nano-robots (ROSrobots). Under the action of a vertical rotating magnetic field, the swarm could climb the slope, cross the obstacle, and quickly reach the other side of the container. Despite the increased slope, the ROSrobot swarm can also move quickly and with high precision (Figure 10f).

## 4. Outlook and Conclusions

Although synthetic micromotors have attracted great interest and been widely investigated during the past two decades, the study of isotropic colloids as active matter and assembly units to construct swarms is just beginning. Isotropic colloids are driven by magnetic dipole–dipole, hydrodynamic, and other forces to form swarms. Here, we focused on some of the well-understood fields/strategies that can lead to collective assembly and are used for motion control. However, faster, more precise, and more programmable strategies for the motion control of swarms are still needed for real applications.

The exploration of micromotor swarms in complex environments is also at an early stage. With the development of applications of micromotor swarms in drug delivery, environmental remediation, sensing, and material science, more real challenging environments will emerge for micromotor swarms to confront. One of the biggest challenges would be the strong fluidic environment in blood vessels. Other challenges include narrow spaces, stents in the vessels, or other implants. For this, the deformation of the swarm as well as path planning are vital for success. For future applications, one challenging task would be how to control micromotor swarms to perform different functions at different stages in a targeted position. More importantly, the development of machine learning is shining new light on active matter. Current progress in this field has been summarized in recent reviews [90,91,92]. The opportunities that machine learning has brought, such as data analysis and the classification of experimental data, come with challenges. Machine learning is a black box to be revealed to promote the development of active matter.

## Data Availability

Not applicable.
